# Metabolite normalization with local radiotherapy following breast tumor resection

**DOI:** 10.1371/journal.pone.0207474

**Published:** 2018-11-16

**Authors:** Meritxell Arenas, Elisabet Rodríguez, Anabel García-Heredia, Salvador Fernández-Arroyo, Sebastià Sabater, Rogelio Robaina, Marina Gascón, Maria Rodríguez-Pla, Noemí Cabré, Fedra Luciano-Mateo, Anna Hernández-Aguilera, Isabel Fort-Gallifa, Jordi Camps, Jorge Joven

**Affiliations:** 1 Department of Radiation Oncology, Hospital Universitari Sant Joan, Institut d’Investigació Sanitària Pere Virgili, Universitat Rovira i Virgili, Reus, Spain; 2 Unitat de Recerca Biomèdica, Hospital Universitari Sant Joan, Institut d’Investigació Sanitària Pere Virgili, Universitat Rovira i Virgili, Reus, Spain; Technische Universitat Munchen, TranslaTUM, GERMANY

## Abstract

The aims of this study were to investigate changes in energy balance-associated metabolites associated with radiotherapy in patients with breast cancer, and to relate these changes to the clinical and pathological response-to-treatment. We studied 151 women with breast cancer who received radiotherapy following surgical excision of the tumor. Blood was obtained before and after the irradiation procedure. The control group was composed of 44 healthy women with a similar age distribution to that of the patients. We analyzed the concentrations of metabolites involved in glycolysis, citric acid cycle and amino acid metabolism using targeted quantitative metabolomics. Post-surgery, pre-radiotherapy, patients had major alterations in the serum concentrations of products of glycolysis, citric acid cycle and amino acid metabolism. The strongest alterations were decreases in serine, leucine and isoleucine concentrations. Alterations in metabolite levels were partially, or totally, reversed after irradiation; the concentrations of serine, leucine and isoleucine approached equivalence to those of the control group. Estrogen receptor-positive patients were those with lower concentrations, while triple negative patients had higher concentrations of these amino acids. The normalization of the amino acids serine, leucine and isoleucine concentrations could be clinically relevant because the normalization of these energy-balance metabolites would suggest that residual micro-metastatic disease had been effectively diminished by the radiotherapy, and may be an indicator of its efficacy.

## Introduction

Breast cancer (BC) is the most frequent type of cancer, and the leading cause of death in women worldwide [1]. To date, the treatment of choice for BC has been surgery followed by loco-regional radiotherapy (RT). In addition, most patients receive adjuvant hormone therapy and/or chemotherapy. The reason for these treatments is to eradicate residual micro-metastatic disease [2]. RT is an effective treatment of BC. However, cancer is heterogeneous, and the sensitivity of patients to treatment is variable. In BC, the efficacy of RT depends on the molecular classification of tumors based on the expression of certain antigens by tumor cells. Some patients with less sensitive phenotypes may require more aggressive oncotherapy with real possibilities of damaging normal tissue adjacent to the cancer target [3]. In many cases, radiation injury could significantly affect patient comfort and effectiveness of the treatment. The metabolic response to RT is largely unknown and this may be clinically relevant considering that recent studies highlight the importance of energy metabolism during oncogenesis. As such, planning and monitoring of RT would be facilitated if the effects of this therapy on tumor cells and the patients’ metabolism were better understood. The new techniques of metabolomics have reinvigorated the hypothesis (formulated in the 1950s by Otto Warburg) that cancer is, fundamentally, a metabolic disease [4]. However, there is a paucity of studies of the metabolome of humans exposed to radiation. Radiation-related changes in small metabolite components of blood are under-researched topics, despite their potential relevance in clinical practice.

The aim of our study was to investigate the changes produced by RT on energy metabolism in patients with BC. Correlating these changes with the clinical and pathological characteristics of patients and tumors would provide an insight into the effectiveness (response to treatment) of the RT.

## Methods

### Participants

We included 151 women (mean age: 55 years, range: 27–85) diagnosed as having BC. They were attending the Department of Radiation Oncology of our Hospital following surgery for the extirpation of the tumor. All patients had a Karnofsky Index >70 and were classified as 0 or 1 on the Eastern Cooperative Oncology Group scale [5]. The exclusion criteria were having previously received RT at the same anatomical site, or to be pregnant or breastfeeding. In the present study, 17 patients received adjuvant chemotherapy post-surgery, 54 patients received adjuvant hormone therapy post-surgery, and 60 received both treatments. The adjuvant chemotherapy treatment duration was for about 4 to 5 months, and concluded 1 to 2 months before RT commencement. The adjuvant hormone treatment commenced 1 to 2 months post-surgery and, usually, was administered simultaneously with RT. The radiation schedule was normofractionated RT (50 Gy at 2 Gy/day on the affected breast and 16 Gy at 2 Gy/day on the tumor bed for 5 days/week). Alternatively, hypofractionated RT was administered at 40 Gy at 2.67 Gy/day for 5 days/week [6, 7]. Some patients received irradiation of regional lymph nodes according to risk factor status [8, 9]. During RT, a weekly acute toxicity assessment was performed using the criteria of the Radiation Therapy Oncology Group and the European Organization for Research and Treatment of Cancer [10]. All patients signed a written informed consent according to the declaration of Helsinki. The study was approved by the Ethics Committee (Institutional Review Board) of the *Hospital Universitari de Sant Joan* (project code: 14/2017).

### Biological samples

Prior to irradiation, and one month following the conclusion of the scheduled RT, blood samples were obtained from the patients. Sera were obtained via centrifugation (2,500 x g, 10 min, 4°C) and stored at –80°C until batched-processed for targeted quantitative metabolomics analyses. The control group was composed of 44 serum samples stored at –80°C in our Biological Sample Bank, and had been obtained from healthy women (mean age: 44 years, range: 27–53) participating in a population-based study conducted in our geographical area. They were ostensibly healthy individuals with no clinical or analytical evidence of infectious disease, renal insufficiency, hepatic damage, neoplasia, oligophrenia, or dementia. A detailed description of this population has been published recently [11].

### Metabolomic analysis of serum samples

We analyzed the concentrations of metabolites involved in glycolysis, citric acid cycle and amino acid metabolism using gas chromatography coupled to quadrupole time-of-flight mass spectrometry with an electron impact source (GC-EI-QTOF-MS), as we have described previously [12]. Analyses were performed with a 7890A gas chromatograph coupled, with an electron impact source, to a 7200 quadrupole time-of-flight mass spectrometer equipped with a 7693 autosampler module and a J&W Scientific HP-5MS column (J&W Scientific HP-5MS column, 30 m × 0.25 mm, 0.25 μm, Agilent Technologies, Santa Clara, CA, USA). Calibration curves were obtained for each metabolite by plotting standard concentrations as a function of the peak area. Recovery of each metabolite was calculated, and ranged between 83% and 99%.

### Molecular classification of tumors

Tumor biopsies were analyzed in the Pathology Laboratory of our Hospital, and classified as follows: The Her2 subtype had a positive expression of human epidermal growth factor receptor 2. The luminal A subtype had positive expression of hormone receptors (estrogens and/or progesterone) and was Her2 negative. The luminal B subtype was positive for the hormone and Her2 receptors, while the triple negative subtype was negative for hormone receptors and Her2.

### Statistical analysis

Data were imported into the MetaboAnalyst 3.0 software package (Mc Gill University, Montreal, Quebec, Canada) for multivariate analysis of pattern recognition, including the unsupervised principal component analysis (PCA) and the supervised partial least squares discriminant analysis (PLSDA). The relative magnitude of observed changes was evaluated using the variable importance in projection (VIP) score [13]. All the other statistical calculations were performed with the statistical package for social sciences (SPSS 22.0, Chicago, IL, USA). Differences between any two groups were assessed with Student’s *t*-test. Pearson correlation coefficient was used to evaluate the degree of association between variables. The diagnostic accuracy of the measured biochemical variables was assessed by receiver operating characteristic (ROC) curves. This analysis represents plots of all the sensitivity/specificity pairs resulting from varying decision thresholds. Sensitivity is the proportion of subjects correctly identified as having a specific disease. Specificity is the proportion of subjects correctly identified as not having a specific disease. False positive rate is calculated as 1-specificity. The area under the curve (AUC) and 95% confidence interval (CI) was calculated. The AUC represents the ability of the test to correctly classify patients according to the investigated alteration. The values of AUC can range between 1 (perfect test) and 0.5 (worthless test) [14].

## Results

### Clinical characteristics of the BC patients

The main clinical variables are shown in Table 1. Patients attending the Department of Radiation Oncology had already undergone surgery for the tumors which, in the majority of cases, were relatively small, without metastasis, with positive estrogen and progesterone receptors and classified, mainly, as luminal A or B. The most common pathological diagnosis was that of invasive ductal carcinoma.

**Table 1:** Clinical characteristics of the breast cancer patients, treated tumors and surgical procedures.

Variable	Characteristics*
Age	55 (14)
Smoking	10.6
Alcohol habit (> 20g/day)	2.0
Arterial hypertension	12.8
Diabetes	3.3
Dyslipidemia	14.1
Chronic obstructive pulmonary disease	1.6
Ischemic heart disease	1.6
Hyperthyroidism	4.6
Menopause state:	
Premenopausal	27.1
Perimenopausal	13.9
Postmenopausal	58.9
Use of oral contraceptives	56.3
Tumor size (TNM system):	
T1	60.9
T2	32.0
T3	6.7
T4	1.3
Nodes (TNM system):	
N0	64.6
N1	28.0
N2	6.0
N3	1.3
Metastasis (TNM system):	
M0	96.0
M1	4.0
Pathological anatomy of the tumor:	
Ductal carcinoma *in situ*	7.9
Lobular carcinoma *in situ*	0.7
Invasive ductal carcinoma	79.5
Invasive lobular carcinoma	1.3
Papillar carcinoma	6.6
Others	4.0
Estrogen receptors:	
Negative	15.3
Positive	84.7
Progesterone receptors:	
Negative	31.3
Positive	68.7
Her2 receptor in tumor biopsy:	
Negative	81.3
Positive	16.7
Tumor molecular classification:	
Luminal A	34.9
Luminal B	36.9
Her2 positive	16.8
Triple negative	11.4
Adjuvant chemotherapy	59.7
Adjuvant hormone therapy	85.3
Neoadjuvant chemotherapy	24.7

*Results are shown in frequencies except for age, which is shown as mean and standard deviation (in parenthesis)

### Metabolite alterations with RT in BC patients

**Fig 1:**
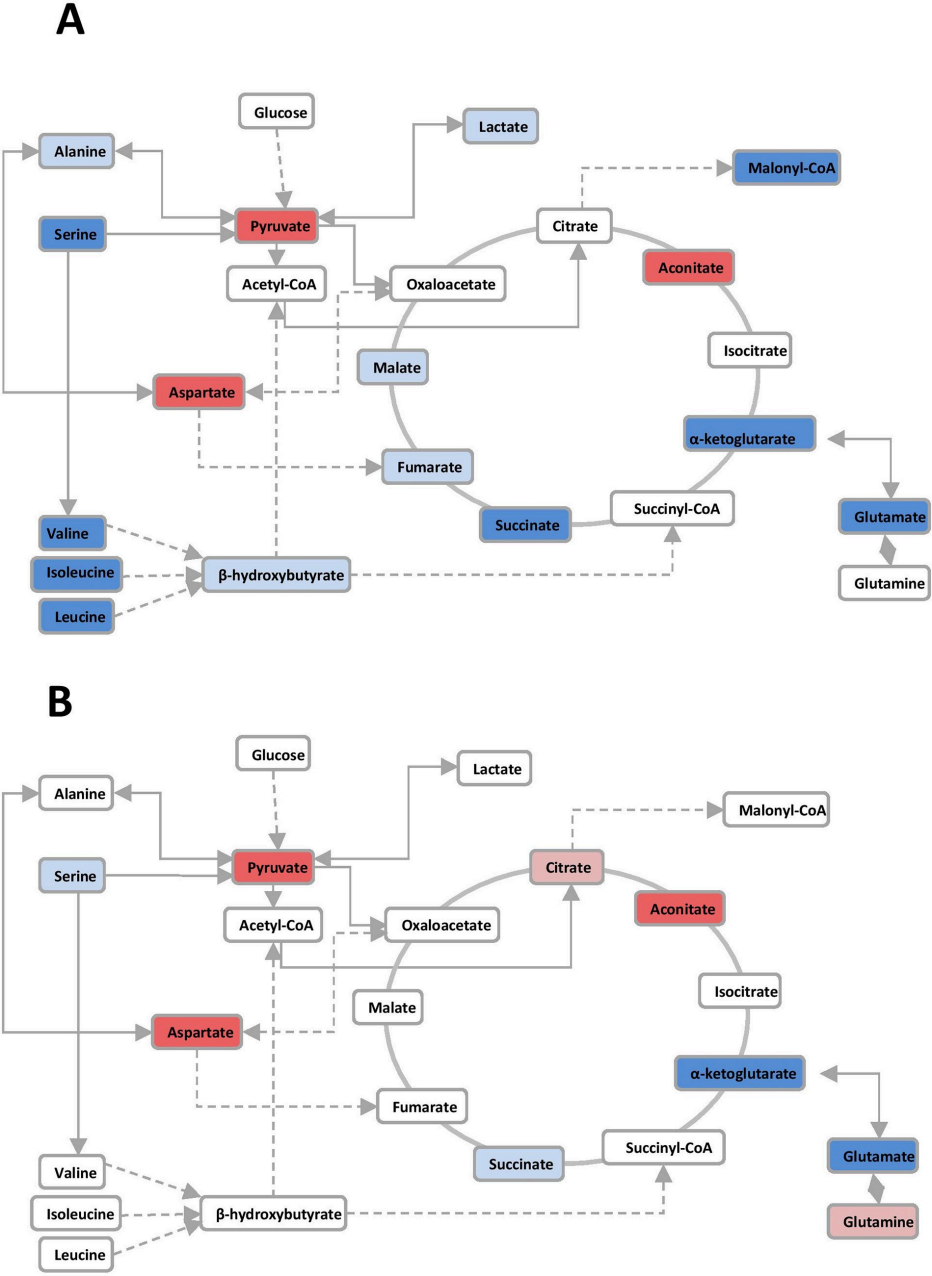
**Alterations in the glycolytic pathway, citric acid cycle, and amino acid pathways in breast cancer patients, relative to control women pre- (A) and post-radiation therapy (B).** Variables that increased are shown in red while those that decreased are shown in blue. The intensity of the color estimates the magnitude of change.

**Fig 2:**
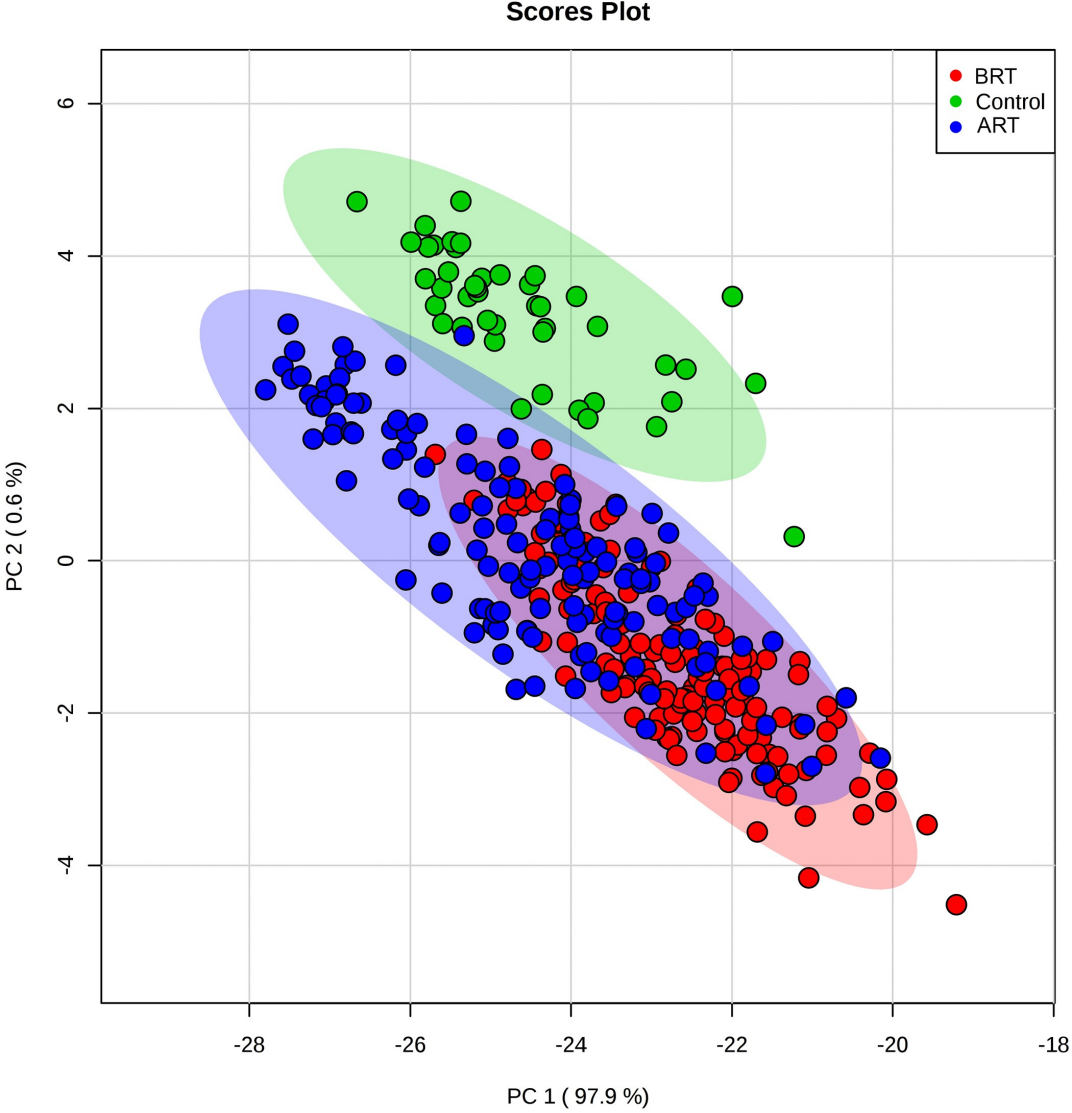
Principal component analysis (PCA) scores. **Plots of serum samples from control women (green) and breast cancer patients pre- (red) and post-radiation therapy (blue);** ART = after radiotherapy; BRT = before radiotherapy.

**Fig 3:**
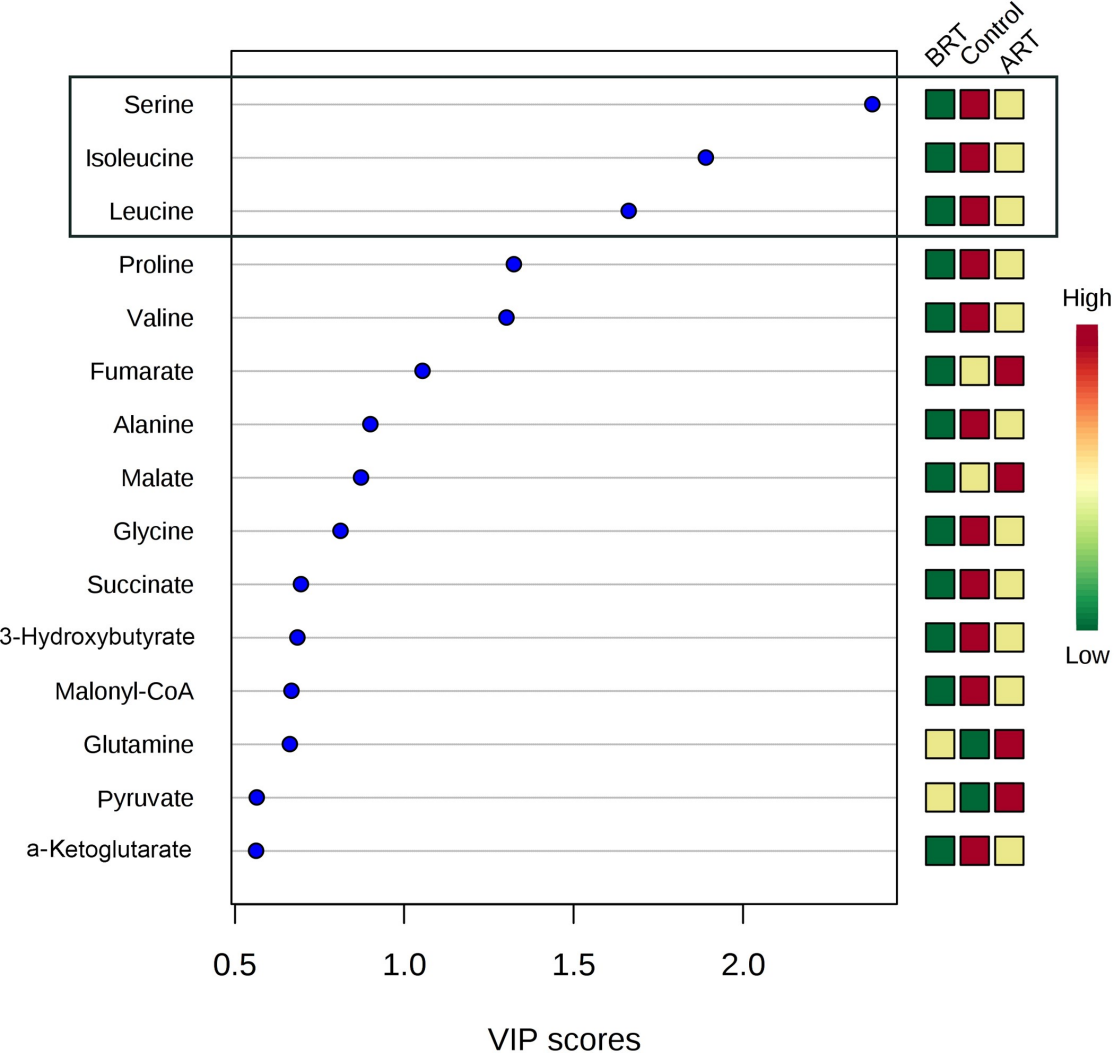
Variable importance in projection (VIP) scores of the partial least squares discriminant analysis (PLSDA). **The most relevant metabolite changes with treatment were those of serine, leucine and isoleucine;** ART = after radiotherapy; BRT: before radiotherapy.

**Fig 4:**
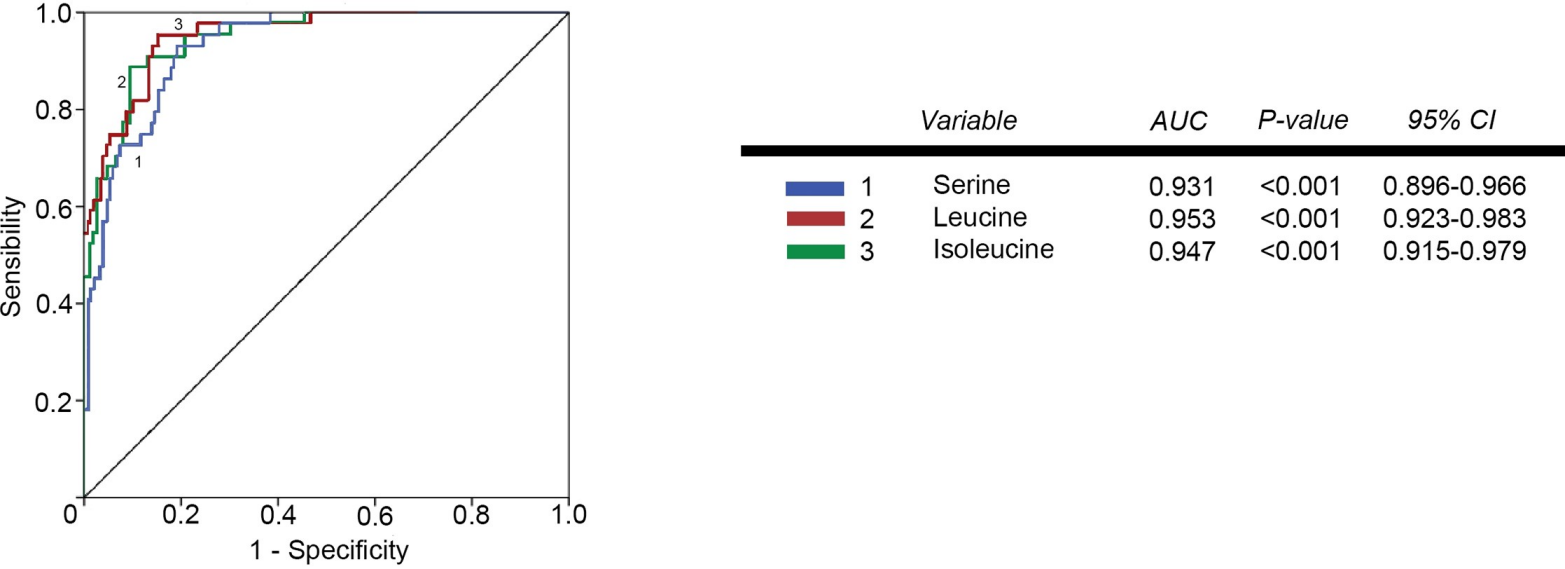
Receiver operating characteristics (ROC) plots of plasma serine, leucine and isoleucine concentrations in breast cancer patients pre-radiotherapy, relative to the control group. AUC = area under the curve.

The individual values of the metabolites analyzed in patients and controls are shown in S1 Table. Before RT, patients had major alterations in the serum concentrations of metabolites involved in glycolysis, citric acid cycle and amino acid metabolism. Parameters having the strongest decreases pre-RT were serine, valine, leucine, isoleucine, succinate, α-ketoglutarate, glutamate and malonyl coenzyme A. The parameters having the strongest increases were pyruvate, aspartate and aconitate. Notably, most of these alterations were completely or partially reversed following RT (Table 2 and Fig 1). The treatment normalized (or almost normalized) the serum concentrations of lactate, alanine, valine, leucine, isoleucine, proline, malonyl coenzyme A, glycine, succinate, serine and ketoglutarate. However, RT was associated with further increases in the levels of pyruvate, fumarate, malate, citrate and glutamine, and did not modify the pre-RT levels of hydroxybutyrate, oxaloacetate, aspartate, glutamate, and aconitate. The score plot of the PCA analysis showed that the control women and the BC patients pre-treatment were, clearly, two distinct populations with little overlap, and that RT produced changes in metabolite levels that tended towards the control group values (Fig 2). To identify the most important metabolites related to changes produced by RT, we derived the VIP score. The score is a measure of the variable’s degree-of-alteration associated with the disease i.e. a higher VIP score is considered more relevant in classification. The VIP analysis identified serine, leucine and isoleucine as the most relevant metabolites (Fig 3). Further, the diagnostic accuracy of these amino acids in discriminating between the control women and the BC patients pre-RT was very high, with AUC’s approaching unity (Fig 4).

**Table 2:** Results of the selected biochemical variables in control women (n = 50) and in breast cancer (BC) patients (n = 150) pre- and post-radiotherapy (RT). Results are shown as means and SD (in parenthesis).

Variable	Control Group	BC Pre-RT	BC Post-RT
Pyruvate (μM)	23.81 (12.07)	58.20 (19.60) ^c^	74.65 (28.61) ^c^^,^^f^
Lactate (μM)	559.46 (62.55)	466.14 (58.37) ^c^	548.27 (112.71) ^f^
Alanine (μM)	199.99 (66.81)	148.97 (50.60) ^c^	211.01 (82.59) ^f^
Hydroxybutyrate (μM)	24.18 (18.07)	22.20 (22.68)	24.72 (21.92)
Valine (μM)	121.68 (45.37)	69.32 (23.38) ^c^	125.03 (72.95) ^f^
Leucine (μM)	68.23 (26.03)	28.85 (11.04) ^c^	59.95 (38.09) ^f^
Isoleucine (μM)	32.77 (15.91)	10.81 (5.41) ^c^	25.09 (18.97) ^a^^,^^f^
Proline (μM)	93.37 (29.73)	42.99 (19.43) ^c^	78.44 (52.87) ^f^
Malonyl Coenzyme A (μM)	1.60 (0.31)	1.08 (0.33) ^c^	1.38 (0.43) ^a^^,^^f^
Glycine (μM)	135.40 (43.91)	78.12 (27.65) ^c^	106.46 (40.34) ^b^^,^^f^
Succinate (μM)	13.79 (4.20)	8.54 (1.97) ^c^	11.14 (2.82) ^c^^,^^f^
Fumarate (μM)	0.37 (0.17)	0.34 (0.17)	0.51 (0.27) ^b^^,^^f^
Serine (μM)	56.51 (20.88)	14.98 (14.23) ^c^	35.71 (30.43) ^c^^,^^f^
Oxaloacetate (μM)	26.56 (7.32)	31.62 (13.16) ^a^	35.91 (11.72) ^c^
Malate (μM)	1.58 (0.76)	1.54 (0.87)	2.14 (1.10) ^a^^,^^e^
Aspartate (μM)	2.07 (0.91)	14.96 (4.95) ^c^	16.01 (6.42) ^c^^,^^e^
Ketoglutarate (μM)	7.48 (9.45)	3.44 (1.62) ^c^	4.30 (2.04) ^c^^,^^e^
Glutamate (μM)	135.47 (45.77)	47.60 (22.25) ^c^	51.48 (22.75) ^c^
Aconitate (μM)	0.12 (0.05)	0.69 (0.62) ^c^	0.55 (0.32) ^c^^,^^d^
Citrate (μM)	33.13 (5.86)	43.61 (8.78) ^c^	54.07 (13.19) ^c^^,^^f^
Glutamine (μM)	36.73 (8.32)	51.50 (12.43) ^c^	68.15 (22.57) ^c^^,^^f^

^a^ *P* < 0.05

^b^ *P* < 0.01

^c^ *P* < 0.001, with respect to the control group

^d^ *P* < 0.05

^e^ *P* < 0.01

^f^ *P* < 0.001, with respect to values Pre-RT.

**Fig 5:**
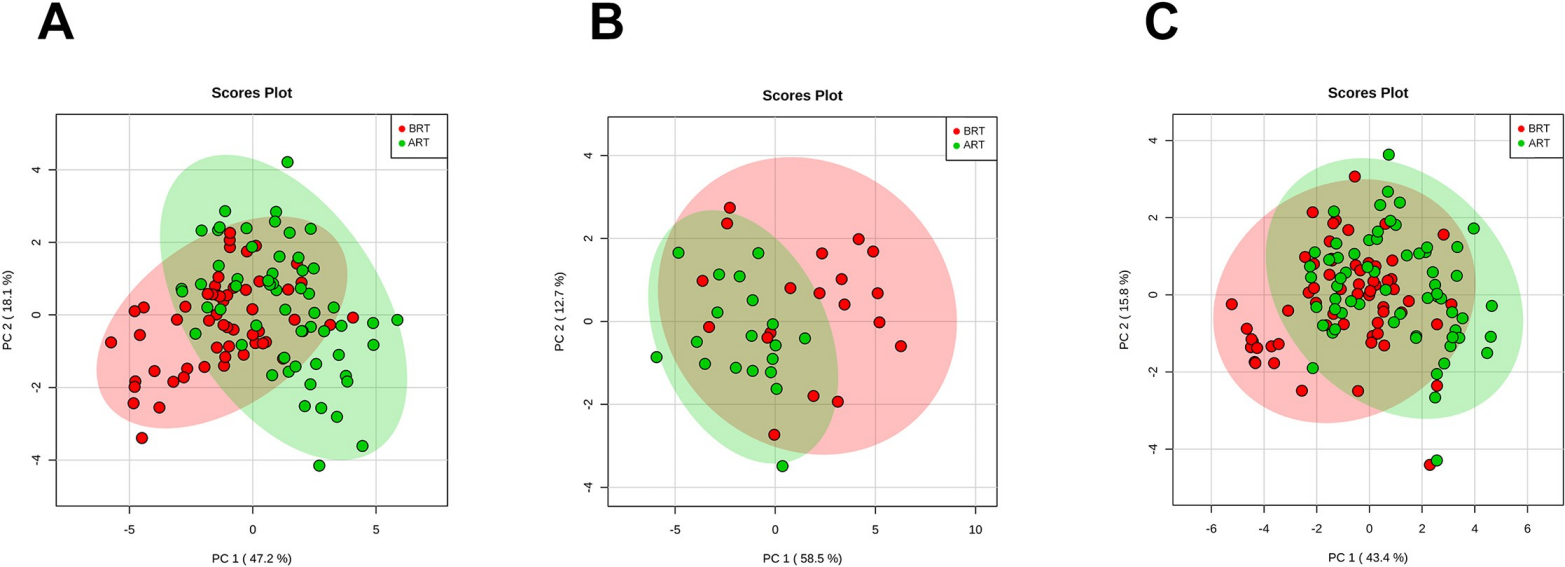
Principal component analysis (PCA) scores. **Plot of serum samples from breast cancer patients who had received hormone therapy (A), chemotherapy (B) or both treatments (C);** ART = after radiotherapy; BRT = before radiotherapy.

**Fig 6:**
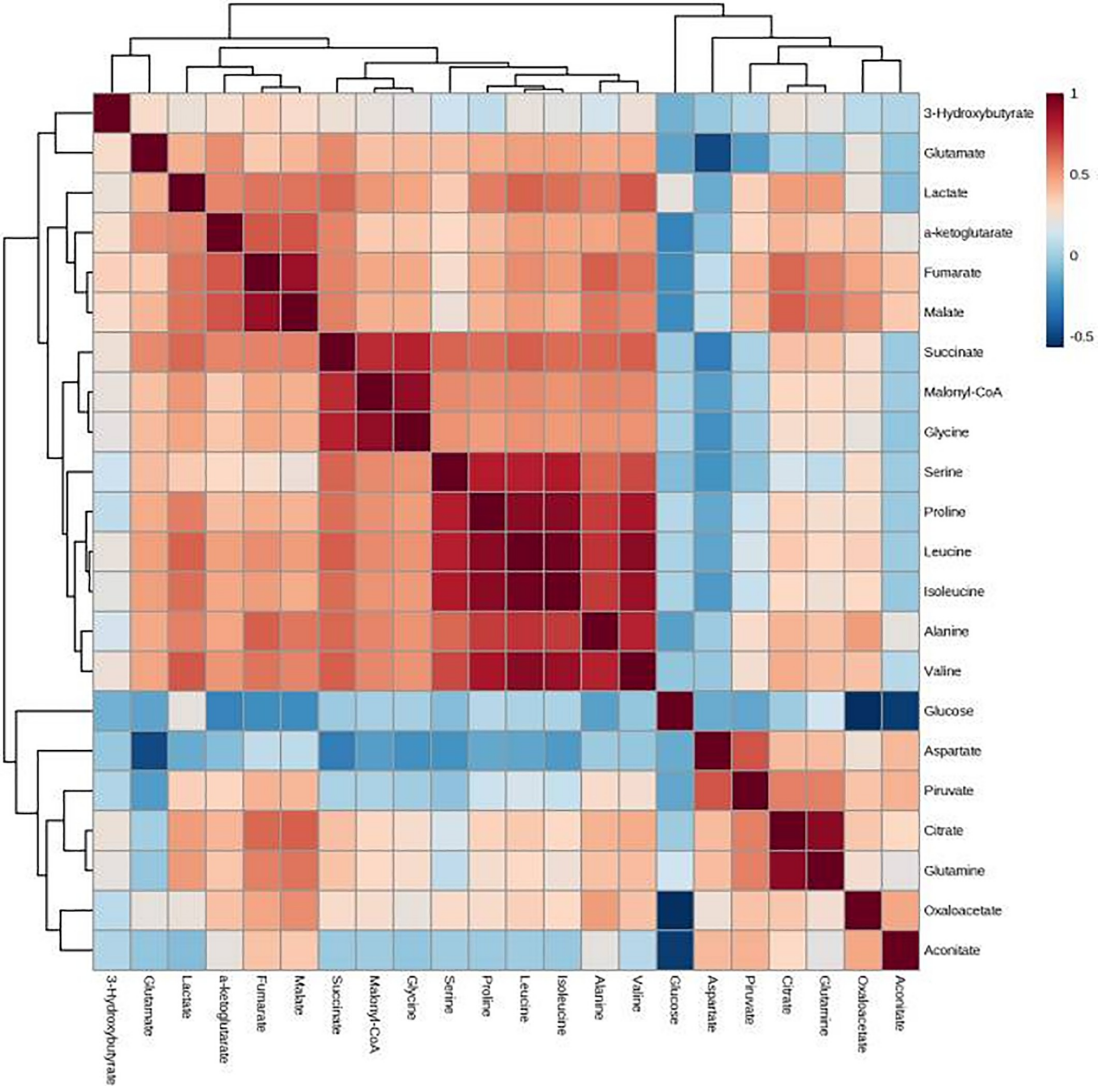
Correlations between the analyzed metabolites. Positive associations are shown in red and negative associations in blue. The intensity of the color estimates the magnitude of change.

Adjuvant hormone therapy was associated with mild changes in serum glycine concentrations post-RT (Table 3). Additionally, adjuvant chemotherapy was associated with mild changes in lactate, oxaloacetate and glutamine concentrations post-RT (Table 4). PCA analysis score plots showed a high degree of overlapping in the serum levels of metabolites in BC patients segregated with respect to whether they had received adjuvant hormone therapy, chemotherapy, or both (Fig 5). Correlations between the different metabolites are shown in Fig 6. The strongest positive associations with RT were with the amino acids.

**Table 3:** Results of the selected metabolites in breast cancer patients post-radiotherapy (RT), and segregated with respect to whether or not they received adjuvant hormone therapy. Results are shown as means and SD (in parenthesis).

Variable	Metabolite concentrations post-RT	
	Hormone therapy NO	Hormone therapy YES
Pyruvate (μM)	84.26 (32.25)	72.42 (27.48)
Lactate (μM)	578.60 (111.08)	541.46 (112.91)
Alanine (μM)	231.06 (92.05)	206.97 (80.33)
Hydroxybutyrate (μM)	20.11 (15.72)	25.91 (23.10)
Valine (μM)	141.59 (75.31)	121.49 (72.50)
Leucine (μM)	68.97 (41.21)	58.07 (37.34)
Isoleucine (μM)	29.93 (21.50)	24.08 (18.33)
Proline (μM)	90.23 (59.08)	76.01 (51.43)
Malonyl Coenzyme A (μM)	1.50 (0.39)	1.36 (0.43)
Glycine (μM)	122.82 (38.32)	103.04 (40.01) *
Succinate (μM)	11.62 (3.01)	11.04 (2.79)
Fumarate (μM)	0.62 (0.33)	0.48 (0.24)
Serine (μM)	39.31 (31.19)	35.03 (30.44)
Oxaloacetate (μM)	36.41 (14.08)	35.70 (11.20)
Malate (μM)	2.59 (1.46)	2.02 (0.97)
Aspartate (μM)	16.10 (7.14)	15.98 (6.30)
Ketoglutarate (μM)	4.78 (2.72)	4.17 (1.84)
Glutamate (μM)	44.84 (24.13)	52.98 (22.38)
Aconitate (μM)	0.50 (0.34)	0.56 (0.31)
Citrate (μM)	57.44 (14.41)	53.38 (12.90)
Glutamine (μM)	75.01 (23.31)	66.83 (22.30)

* *P* < 0.05

**Table 4:** Results of the selected metabolites in breast cancer patients post-radiotherapy (RT), and segregated with respect to whether or not they received adjuvant chemotherapy. Results are shown as means and SD (in parenthesis).

Variable	Metabolite concentrations post-RT	
	Chemotherapy NO	Chemotherapy YES
Pyruvate (μM)	79.61 (26.09)	70.70 (30.23)
Lactate (μM)	570.20 (115.75)	530.86 (108.52) *
Alanine (μM)	215.12 (83.98)	208.37 (82.23)
Hydroxybutyrate (μM)	23.71 (18.25)	25.72 (24.67)
Valine (μM)	127.93 (72.22)	123.07 (74.32)
Leucine (μM)	60.27 (37.35)	59.95 (39.06)
Isoleucine (μM)	25.97 (19.64)	14.54 (18.61)
Proline (μM)	79.66 (53.78)	77.85 (52.71)
Malonyl Coenzyme A (μM)	1.43 (0.44)	1.35 (0.42)
Glycine (μM)	107.55 (41.41)	106.23 (39.72)
Succinate (μM)	11.35 (2.83)	10.98 (2.84)
Fumarate (μM)	0.51 (0.27)	0.49 (0.26)
Serine (μM)	35.69 (31.44)	35.94 (29.96)
Oxaloacetate (μM)	33.59 (10.41)	37.67 (12.47) *
Malate (μM)	2.10 (1.01)	2.14 (1.16)
Aspartate (μM)	16.51 (6.95)	15.56 (5.98)
Ketoglutarate (μM)	4.34 (1.86)	4.24 (2.17)
Glutamate (μM)	54.01 (21.88)	49.44 (23.54)
Aconitate (μM)	0.51 (0.29)	0.57 (0.34)
Citrate (μM)	55.51 (10.99)	52.96 (14.82)
Glutamine (μM)	73.26 (21.02)	63.87 (23.19) *

* *P* < 0.05

### Relationships between selected amino acids and the patients’ characteristics

**Fig 7:**
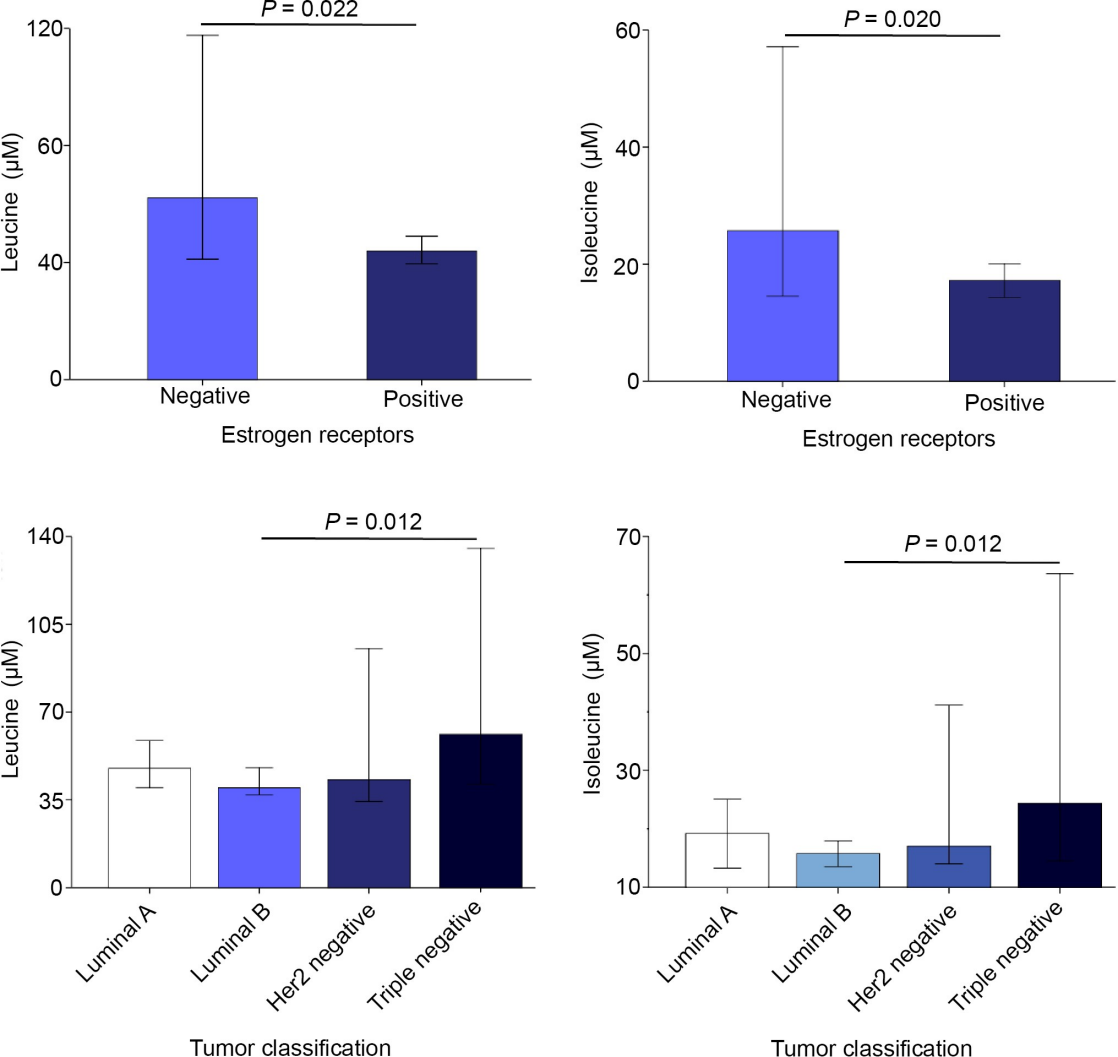
Serum leucine and isoleucine concentrations post-RT in breast cancer patients segregated with respect to presence of estrogen receptors and tumor classification.

We assessed whether the concentrations of the most severely altered metabolites (the amino acids serine, leucine and isoleucine) were related to the characteristics of the tumors, or the toxicological response of the patients. We found that serum concentrations of leucine and isoleucine post-RT were significantly lower in estrogen-receptor-positive patients than in estrogen-receptor-negative patients and higher in triple-negative patients relative to luminal and Her2 subgroups (Fig 7). We did not find any significant associations between the serum concentrations of these metabolites and any other tumor characteristic, or to toxicological response (S2 Table).

## Discussion

To the best of our knowledge there have been no published data on changes in energy metabolism in BC post-RT. The present findings highlight striking alterations in the serum concentrations of metabolites in BC patients post-surgery; the RT being associated with modifications of these levels such that there was a trend towards levels associated with the healthy (control) women. One of the questions that arise from these data is whether this improvement depends on RT *per se* or is influenced by the concomitant treatment with chemotherapy or hormone therapy that many of our patients had had. Our results indicate that the effects of these adjuvant treatments are quite small, which leads us to believe that the apparent normalization of the metabolic profile is due to the RT. Another possibility is that metabolite levels tend to normalize due, simply, to the passage of time post-surgery, and not as a direct action of the RT. This hypothesis cannot be realistically ruled out, since a group of BC patients not receiving RT could not be studied for obvious ethical reasons. However, recent studies also showed effects of RT on the metabolism of experimental animals and patients with various other types of cancer. Indeed, in accord with the results obtained in the present investigation, RT has been shown to increase the fecal content of α-ketobutyrate, valine, isoleucine, and alanine in cervical cancer patients [15]. RT also influenced the serum concentrations of 28 metabolites in patients with glyoma [16], increased the hepatic glutamine concentrations [17], and significantly modified the saliva metabolome [18] in mice. Taken together, these data suggest that RT does, indeed, influence metabolism in cancer patients.

The partial normalization of energy metabolism after RT may have positive effects on the clinical evolution of patients, given the implications of alterations in the levels of various metabolites with cancer development and its associated complications. Various studies have shown that the availability of circulating amino acids is often reduced in cancer patients [19, 20]. A major finding of the present study is the important decrease in the circulating levels of serine, leucine and isoleucine pre-RT. This could be interpreted as an enhanced cellular demand for these metabolites. The metabolism of most cancers relies on the use of aerobic glycolysis and glutamine catabolism to support cancer cell growth [21–23]. Glutamine is taken-up by cancer cells, and is involved in the replenishment of the mitochondrial citric acid carbon pool, which is required for the synthesis of lipids, proteins, and nucleic acids. The high demand for glutamine leads to an increased uptake by the tumor cells of serine and branched-chain amino acids (BCAA), including leucine and isoleucine. These amino acids provide an important source for the biosynthesis of glutamine and its metabolite derivative, glutamate. The increased demand on glutamine would explain the reduced serum concentrations of serine and BCAA in actively replicating tumor cells.

One of the most important consequences of cancer is cachexia. This is a major contributing cause of mortality in up to 50% of patients with severe cancer [24]. Cachexia is characterized by adipose and skeletal muscle tissue loss. This reduces the quality of life and the efficacy of many chemotherapeutic interventions. The mechanisms involved in the development and progression of cachexia are complex, but alterations in BCAA metabolism play a fundamental role. Under normal conditions, BCAA oxidation in skeletal muscle provides approximately 7% of the energy needs, but under highly catabolic circumstances such as cancer cachexia, the contribution can be as high as 20% [25]. In this context, the finding that the circulating levels of BCAA are reduced in BC patients (even several weeks after surgery), and that this alteration may be reversed by RT may have special clinical relevance.

We found an association between the expression of estrogen receptors by the tumors and the concentrations of leucine and isoleucine post-RT. Patients with positive estrogen receptors had lower levels and triple-negative patients had higher levels of these two BCAAs. The relationships between the molecular subtypes of BC and energy metabolism have not been well characterized, to date. Patients with triple-negative BC have been reported to exhibit higher levels of glycolysis and higher expression of the glucose transporter protein [26], together with lower levels of glutamine and higher levels of glutamate [27]. We did not find any significant differences in the serum concentrations of metabolites pre-RT with respect to the molecular classification of tumors. However, of note is that we did find differences in their serum concentrations post-RT, indicating significant differences in the metabolic response to treatment. Explanations for this finding cannot be derived from the present investigation. However, our observation may open new lines of enquiry that, potentially, could be useful in better understanding the differences between tumor subtypes and, hence, therapy selection.

## Conclusion

The results of the present study showed that the serum concentrations of products of glycolysis, citric acid cyclic and amino acid metabolism are considerably altered in women with BC, even several weeks post-surgery, and that RT is associated with a partial rectification of these alterations. The normalization of serine and BCAA concentrations is of considerable note since these amino acids are crucial in energy metabolism and the metabolic consequences of cancer. Our report represents an early application of metabolomics in the study of breast cancer and the consequences of treatment such as radiotherapy. Further investigation on a wider series of patients and in women pre- and post-surgery, is needed to increase our knowledge of the metabolic alterations associated with this disease, and the efficacy of the treatments available for tumor control.

## Supporting information

S1 Table Supplementary Table 1. (SAV)

S2 Table Supplementary Table 2. (DOC)

## References

[1] Peairs KS, Choi Y, Stewart RW, Sateia HF. Screening for breast cancer. Semin Oncol. 2017; 44: 60–72. 10.1053/j.seminoncol.2017.02.004 28395765 10.1053/j.seminoncol.2017.02.004

[2] Tenori L, Oakman C, Morris PG, Gralka E, Turner N, Cappadona S, et al Serum metabolomic profiles evaluated after surgery may identify patients with oestrogen receptor negative early breast cancer at increased risk of disease recurrence. Results from a retrospective study. Mol Oncol. 2015; 9:128–139. 10.1016/j.molonc.2014.07.012 25151299 10.1016/j.molonc.2014.07.012 PMC5528693

[3] Jelonek K, Pietrowska M, Widlak P. Systemic effects of ionizing radiation at the proteome and metabolome levels in the blood of cancer patients treated with radiotherapy: the influence of inflammation and radiation toxicity. Int J Radiat Biol. 2017; 93: 683–696. 10.1080/09553002.2017.1304590 28281355 10.1080/09553002.2017.1304590

[4] Warburg O. On the origin of cancer cells. Science. 1956;123: 309–314. 13298683 10.1126/science.123.3191.309

[5] Sloan JA, Loprinzi CL, Laurine JA, Novotny PJ, Vargas-Chanes D, Krook JE. A simple stratification factor prognostic for survival in advanced cancer: the good/bad/uncertain index. J Clin Oncol. 2001;19: 3539–3546. 10.1200/JCO.2001.19.15.3539 11481361 10.1200/JCO.2001.19.15.3539

[6] Montero A, Sanz X, Hernanz R, Cabrera D, Arenas M, Bayo E, et al Accelerated hypofractionated breast radiotherapy: FAQs (frequently asked questions) and facts. Breast 2014; 23: 299–309. 10.1016/j.breast.2014.01.011 24530095 10.1016/j.breast.2014.01.011

[7] Prades J, Algara M, Espinàs JA, Farrús B, Arenas M, Reyes V, et al Understanding variations in the use of hypofractionated radiotherapy and its specific indications for breast cancer: A mixed-methods study. Radiother Oncol. 2017; 123: 22–28. 10.1016/j.radonc.2017.01.014 28236538 10.1016/j.radonc.2017.01.014

[8] Arenas M, Montero Á, de Las Peñas MD, Algara M. The position and current status of radiation therapy after primary systemic therapy in breast cancer: a national survey-based expert consensus statement. Clin Transl Oncol. 2016; 18: 582–591. 10.1007/s12094-015-1401-0 26370420 10.1007/s12094-015-1401-0

[9] Aristei C, Kaidar-Person O, Arenas M, Coles C, Offersen BV, Bourgier C, et al The 2016 Assisi Think Tank Meeting on breast cancer: white paper. Breast Cancer Res Treat. 2016; 160: 211–221. 10.1007/s10549-016-3998-2 27686462 10.1007/s10549-016-3998-2

[10] Bots WTC, van den Bosch S, Zwijnenburg EM, Dijkema T, van den Broek GB, Weijs WLJ, et al Reirradiation of head and neck cancer: Long-term disease control and toxicity. Head Neck. 2017; 39: 1122–1130. 10.1002/hed.24733 28263446 10.1002/hed.24733 PMC5485062

[11] Fort-Gallifa I, García-Heredia A, Hernández-Aguilera A, Simó JM, Sepúlveda J, Martín-Paredero V, et al Biochemical indices of oxidative stress and inflammation in the evaluation of peripheral artery disease. Free Radic Biol Med. 2016; 97: 568–576. 10.1016/j.freeradbiomed.2016.07.011 27449545 10.1016/j.freeradbiomed.2016.07.011

[12] Riera-Borrull M, Rodríguez-Gallego E, Hernández-Aguilera A, Luciano F, Ras R, Cuyàs E, et al Exploring the process of energy generation in pathophysiology by targeted metabolomics: Performance of a simple and quantitative method. J Am Soc Mass Spectrom. 2016; 27: 168–177. 10.1007/s13361-015-1262-3 26383735 10.1007/s13361-015-1262-3

[13] Grootveld M. Introduction to the applications of chemometric techniques in ‘Omics’ research: Common pitfalls, misconceptions and ‘rights and wrongs’. In: Grootveld M, editor. Metabolic Profiling: Disease and Xenobiotics. Cambridge: Royal Society of Chemistry; 2014 pp 1–34.

[14] Zweig MH, Campbell G. Receiver-operating characteristics (ROC) plots: a fundamental evaluation tool in clinical medicine. Clin Chem. 1993; 39: 561–577. 8472349

[15] Chai Y, Wang J, Wang T, Yang Y, Su J, Shi F, et al Application of 1H NMR spectroscopy-based metabolomics to feces of cervical cancer patients with radiation-induced acute intestinal symptoms. Radiother Oncol. 2015; 117: 294–301. 10.1016/j.radonc.2015.07.037 26277430 10.1016/j.radonc.2015.07.037

[16] Mörén L, Wibom C, Bergström P, Johansson M, Antti H, Bergenheim A. Characterization of the serum metabolome following radiation treatment in patients with high-grade gliomas. Radiat Oncol. 2016; 11: 51 10.1186/s13014-016-0626-6 27039175 10.1186/s13014-016-0626-6 PMC4818859

[17] Xiao X, Hu M, Zhang X, Hu JZ. NMR-based metabolomics analysis of liver from C57BL/6 mouse exposed to ionizing radiation. Radiat Res. 2017; 188:44–55. 10.1667/RR14602.1 28463589 10.1667/RR14602.1 PMC5564182

[18] Laiakis EC, Strawn SJ, Brenner DJ, Fornace AJ Jr. Assessment of saliva as a potential biofluid for biodosimetry: A pilot metabolomics study in mice. Radiat Res. 2016; 186:92–7. 10.1667/RR14433.1 27332953 10.1667/RR14433.1 PMC5714587

[19] Asiago VM, Alvarado LZ, Shanaiah N, Gowda GA, Owusu-Sarfo K, Ballas RA, et al Early detection of recurrent breast cancer using metabolite profiling. Cancer Res. 2010; 70: 8309–8318. 10.1158/0008-5472.CAN-10-1319 20959483 10.1158/0008-5472.CAN-10-1319 PMC2995269

[20] Miyagi Y, Higashiyama M, Gochi A, Akaike M, Ishikawa T, Miura T, et al Plasma free amino acid profiling of five types of cancer patients and its application for early detection. PLoS One. 2011; 6: e24143 10.1371/journal.pone.0024143 21915291 10.1371/journal.pone.0024143 PMC3168486

[21] Vander Heiden MG, Cantley LC, Thompson CB. Understanding the Warburg effect: the metabolic requirements of cell proliferation. Science. 2009; 324: 1029–1033. 10.1126/science.1160809 19460998 10.1126/science.1160809 PMC2849637

[22] Hensley CT, Wasti AT, DeBerardinis RJ. Glutamine and cancer: cell biology, physiology, and clinical opportunities. J Clin Invest. 2013; 123: 3678–3684. 10.1172/JCI69600 23999442 10.1172/JCI69600 PMC3754270

[23] Mishra P, Ambs S. Metabolic signatures of human breast cancer. Mol Cell Oncol. 2015; 2: pii: e992217,2015 10.4161/23723556.2014.992217 26005711 10.4161/23723556.2014.992217 PMC4438683

[24] Dewys WD, Begg C, Lavin PT, Band PR, Bennett JM, Bertino JR, et al Prognostic effect of weight loss prior to chemotherapy in cancer patients. Eastern Cooperative Oncology Group. Am J Med. 1980; 69: 491–497. 7424938 10.1016/s0149-2918(05)80001-3

[25] Lam VW, Poon RT. Role of branched-chain amino acids in management of cirrhosis and hepatocellular carcinoma. Hepatol Res. 2008; 38: S107–S115. 10.1111/j.1872-034X.2008.00435.x 19125941 10.1111/j.1872-034X.2008.00435.x

[26] Long JP, Li XN, Zhang F. Targeting metabolism in breast cancer: How far we can go? World J Clin Oncol. 2016; 7: 122–130. 10.5306/wjco.v7.i1.122 26862496 10.5306/wjco.v7.i1.122 PMC4734934

[27] Cao MD, Lamichhane S, Lundgren S, Bofin A, Fjøsne H, Giskeødegård GF, et al Metabolic characterization of triple negative breast cancer. BMC Cancer. 2014; 14: 941 10.1186/1471-2407-14-941 25495193 10.1186/1471-2407-14-941 PMC4295321

