# Supplementary material for: Metabolite normalization with local radiotherapy following breast tumor resection
Source: PLoS One. 2018 Nov 16;13(11):e0207474. doi: 10.1371/journal.pone.0207474 (PMC6239311; doi:10.1371/journal.pone.0207474)
Supplement: S2 Table — (DOC) [file pone.0207474.s002.doc]

**Supplementary Table 2**

**Supplementary Table 2a:** Associations between selected amino acid concentrations pre-radiotherapy, tumor characteristics, and toxic reactions to treatment (*P* values by ANOVA).

|  | **Tumor characteristics** | | | Toxic reactions | | |
| --- | --- | --- | --- | --- | --- | --- |
| Metabolite | Tumor size | Nodes | **Metastasis** | **Dermatitis** | **Breast pain** | **Asthenia** |
| Leucine (μM) | 0.633 | 0.901 | 0.266 | 0.149 | 0.565 | 0.181 |
| Isoleucine (μM) | 0.497 | 0.814 | 0.273 | 0.315 | 0.486 | 0.299 |
| Serine (μM) | 0.293 | 0.568 | 0.081 | 0.990 | 0.076 | 0.303 |

**Supplementary Table 2b:** Associations between selected amino acid concentrations post-radiotherapy, tumor characteristics, and toxic reactions to treatment (*P* values by ANOVA).

|  | **Tumor characteristics** | | | Toxic reactions to treatment | | |
| --- | --- | --- | --- | --- | --- | --- |
| Metabolite | Tumor size | Nodes | **Metastasis** | **Dermatitis** | **Breast pain** | **Asthenia** |
| Leucine (μM) | 0.222 | 0.637 | 0.054 | 0.216 | 0.052 | 0.194 |
| Isoleucine (μM) | 0.296 | 0.714 | 0.760 | 0.319 | 0.053 | 0.189 |
| Serine (μM) | 0.119 | 0.493 | 0.187 | 0.379 | 0.075 | 0.432 |
